# Ventilation Diagnosis of Angle Grinder Using Thermal Imaging

**DOI:** 10.3390/s21082853

**Published:** 2021-04-18

**Authors:** Adam Glowacz

**Affiliations:** Department of Automatic Control and Robotics, Faculty of Electrical Engineering, Automatics, Computer Science and Biomedical Engineering, AGH University of Science and Technology, 30-059 Kraków, Poland; adglow@agh.edu.pl

**Keywords:** fault detection, angle grinder, power tool, thermal images, diagnosis, image processing

## Abstract

The paper presents an analysis and classification method to evaluate the working condition of angle grinders by means of infrared (IR) thermography and IR image processing. An innovative method called BCAoMID-F (Binarized Common Areas of Maximum Image Differences—Fusion) is proposed in this paper. This method is used to extract features of thermal images of three angle grinders. The computed features are 1-element or 256-element vectors. Feature vectors are the sum of pixels of matrix **V** or PCA of matrix **V** or histogram of matrix **V**. Three different cases of thermal images were considered: healthy angle grinder, angle grinder with 1 blocked air inlet, angle grinder with 2 blocked air inlets. The classification of feature vectors was carried out using two classifiers: Support Vector Machine and Nearest Neighbor. Total recognition efficiency for 3 classes (*TR_AG_*) was in the range of 98.5–100%. The presented technique is efficient for fault diagnosis of electrical devices and electric power tools.

## 1. Introduction

The use of power tools (electric impact drills, angle grinders, cordless screwdrivers, etc.) can be found in the construction industry. Faults of electric power tools can be dangerous for employees’ health. Condition monitoring enables early detection of machine faults. Highly effective diagnosis techniques of electric power tools can decrease the number of accidents and economical losses. For these reasons investigation of fault diagnosis of electric power tools is essential. The development of new methods and techniques of fault diagnosis is profitable. Fault diagnosis techniques can be focused on various parts of equipment for example bearings, ventilation, gears, brushes, stator faults, rotor faults, shaft, etc. The development of fault diagnosis techniques is also related to signal analysis for example: acoustic and vibration signals.

In this paper, the fault diagnosis technique of angle grinders is shown. The proposed technique is based on the processing of thermal images. The author of the paper developed an original method of image processing−BCAoMID−F (Binarized Common Areas of Maximum Image Differences−Fusion). The BCAoMID−F extracted feature vectors from thermal images. The computed feature vectors are 1-element or 256-element vectors. Feature vectors are the sum of pixels of matrix **V** or PCA of matrix **V** or histogram of matrix **V**. Support Vector Machine and Nearest Neighbor classified feature vectors. The paper has the following sections: (1) Introduction, (2) Theoretical background (3) Processing and recognition of the thermal image (4) Analyzed states of the angle grinder (5) Results of the analysis of thermal images (6) Conclusions.

## 2. Theoretical Background

Many various techniques can be applied for fault diagnosis of electrical power tools and electric motors. The first group of diagnostic techniques is based on the analysis of electrical currents. Electrical currents are good diagnostic signals. Differences between FFT spectra of healthy motor and faulty motor are significant. However, analysis of electrical currents is limited to several types of faults, namely: broken bars, shorted coils, overvoltages. Several methods for the analysis of electrical currents have been suggested [1,2].

There are several techniques of non-invasive fault diagnosis, namely: acoustic analysis, vibration analysis, thermal imaging, analysis of ultrasounds. Vibration and acoustic signals are a valuable source of information about electric motors. The sounds and vibrations of a motor carry information about its operating condition. Moreover, vibration and acoustic signals of the motor usually change when a failure occurs.

Areas, where numerous acoustic applications have been published, are fault diagnosis of materials, motors, engines, storage tanks, pipes. The acoustic analysis has some pros and cons. Acoustic analysis is useful for the detection of defects (faulty motor, device). Advantages of acoustic analysis are non-destructive testing, high recognition efficiency, and short analysis time. However, acoustic signals should be captured properly. Captured acoustic signals are influenced by a microphone, environmental conditions, reflected acoustic signals, different parameters of recording software. Several techniques for acoustic fault diagnosis have been proposed [3,4,5,6,7,8,9,10].

An early fault detection method of planetary gearbox was developed. It was based on the acoustic emission technique. It used improved variational mode decomposition for fault detection. Different working conditions (normal condition, crack fault in sun gear, crack fault in planet gear, crack fault in ring gear) were distinguished using values of energy entropy. Experiments were carried out using an experimental setup of a planetary gearbox [3]. An acoustics-based noncontact diagnosis method based on a microphone array was developed. The authors of the paper used the broadband weighted multiple signal classification method and Levenberg-Marquardt and Crank Nicolson method. The accuracy of the location of the faulty wheel was proper for the proposed method [4]. Three different types of acoustic emission sensors (R6a, WSa, Pico) were used for fault diagnosis of ball bearings. Classification of different four states was carried out using a neural network. The results showed that the R6a sensor was the best for acoustic analysis [5]. Fault detection of ball bearing using acoustic signals was developed. PCA, FDA methods, and Adaptive Neuro-Fuzzy Inference System were used. The ANFIS accuracy was equal to 100% for the first principal component [6]. An acoustical damage detection method of the yaw system was described. It was based on a Bayesian network and a three-layer neural network. The computed accuracy was equal to 100% [7]. An acoustic-based fault diagnosis method was developed. It was used for induction motor. The Shortened Method of Frequencies Selection (SMoFS-10) and LSVM were used for fault diagnosis. The recognition rate was in the range of 90–100% [8]. An extended method SMOFS-25-EXPANDED was also developed. The total efficiency of recognition of acoustic signal was equal to 99% for loaded synchronous motor and NN classifier [9]. Fault diagnosis of angle grinders and electric impact drills using acoustic signals and SMOFS-NFC was presented in the literature. The classification accuracy was between 90.66–100% for three angle grinders [10].

Vibration analysis is widely used to detect faulty gears and bearings faults. It is also used for the detection of broken bars of induction motors. Another area where vibration fault diagnosis is used: rolling element defects, shaft defects, gearbox, freewheels, fans, drive-trains, pumps, bridges, pipes, turbine blades. Vibration analysis has some pros and cons. It is non-invasive. Vibration analysis enables real-time monitoring. There are well-developed signal processing methods. However, there are some disadvantages. Localization of fault is difficult. It is difficult to detect some types of faults e.g., isolation faults, slightly shorted coils. Vibration analysis is described well in the literature [11,12,13,14].

The wind turbine gearbox was analyzed. The authors of the paper analyzed speed, vibration, and acoustic signal. Multi-variable feature data set was formed. Wavelet transform was used to extract the statistical features of signals. Classification accuracy of 92% was computed for adaptive neuro-fuzzy inference system (ANFIS) [11]. A distribution-invariant deep belief network (DIDBN) was proposed for vibration-based fault diagnosis of machines. Two diagnosis cases were analyzed by the authors. Raw vibration data was used. The results proved that DIDBN is able to learn distribution-invariant features. Computed recognition accuracies were higher than 96% for all the five analyzed states: fault on the bearing of the planet gear, tooth crack on the planet gear, flake on the sun gear, wear on the sun gear, normal condition [12]. A multi-sensor approach was developed for fault diagnosis of mechanical faults of high voltage circuit breakers. The multi-sensor approach was based on the analysis of vibration signals. The authors of the paper developed an improved multi-sensor evidence combined rule. The efficiency of the developed approach was proved [13]. A technique of analysis of the motor vibration signals of bearing faults of the induction motor was proposed. The proposed method used a high-frequency signal from a microwave sensor. Rational Dilation Wavelet Transforms (RDWT) were used for the following faults: full-load, no-load, rotor bar faults. The recognition accuracy was equal to 93% [14].

Next, a technique of non-destructive testing of motors is thermal imaging. Areas where research and applications of thermal imaging are the following: veterinary, medical applications, security, energy efficiency, mechanical maintenance, electrical maintenance. Thermal imaging is used for the detection of thermal anomalies on the surface of the motor. Thermal imaging can detect many electrical faults, namely: broken bars, shorted coils, insulation faults, fan faults, overvoltages, ventilation faults. Analysis of thermal images can detect the type and location of fault [15,16,17,18,19,20,21,22,23].

An infrared camera was used for fault diagnosis of the three-phase induction motor. Thermal images were captured every second. The analyzed motor was mounted on a base plate with an alignment device. The presented results proved the usefulness of thermal-based fault diagnosis [15]. A convolutional neural network (CNN) and thermal images were used for fault diagnosis of a gearbox. Online remote monitoring was implemented. The recognition rate achieved nearly 100%. Following faults were analyzed: breakages, tooth pitting, cracks [16]. The authors of the paper presented thermal condition monitoring of the three-phase induction motors. It was used for the detection of bearings faults such as inner race, outer race, and ball bearing defects. A new color model using HSV was applied for the proposed method. Next Roberts, Prewitt, Sobel, Canny filters were used for segmenting the Hue component. Next Skewness, Standard Deviation, Kurtosis, Mean, Mean Square Error, Variance, Peak Signal to Noise Ratio were computed. The results indicated that the proposed image segmentation methods and metrics were useful for image recognition [17]. The analysis of the specific region of thermal images of the induction motor was developed. The analysis was performed for the following faults of the motor: with different supply frequencies, with a mechanical load. The results indicated that the analysis of areas is good enough for detecting bearing fault [18]. An approach to detecting mechanical faults of the induction motor using thermography was described. The proposed approach was verified by many experiments in the industry. It proved that analysis of thermal images is useful for fault diagnosis of induction motor [19]. The authors proposed an automatic bearing fault diagnosis approach of three-phase induction motor. The proposed approach was based on thermal infrared imaging. Following classes were analyzed: healthy bearing, outer inner race defected bearing, race defected bearing, lack of lubrication. The authors used 2D-DWT and SVM. The computed results showed high recognition results for bearing fault detection [20]. The self-heating effects of piezoceramic flexible patches were analyzed in the paper. A thermocouple was used for temperature measurement. Next, the authors assessed how the temperature, produced by self-heating of the piezoceramic, is influenced by the surrounding material [21]. The freshness of eggs was analyzed using pulsed thermography and image processing method such as a morphological operator with ‘white top hat’. The proposed approach was highly effective for quality control tools [22]. The surface temperature of lithium-ion polymer cells using infrared thermography was analyzed. The analysis focused on surface temperature distribution for different discharging rates of lithium-ion polymer cells. The analysis proved that infrared thermography analysis has higher accuracy than analysis based on thermocouples [23]. A short review of fault diagnosis methods was presented in [24].

## 3. Processing and Recognition of the Thermal Image

**Figure 1:**
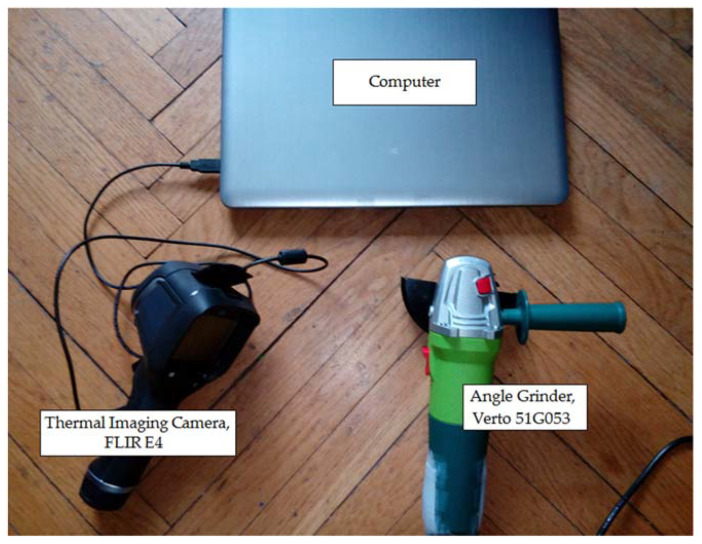
Measurement of the thermal image of the angle grinder (Verto 51G053).

Measurements were carried out in a room of 3 m × 3 m. The temperature of the surface was different for each state of the inspected angle grinder. To measure the temperature of the surface, the author used FLIR E4 thermal imaging camera (range of temperature measurement <−20 °C, +250 °C>, thermal sensitivity less than 0.15 °C, image frequency equal to 9 Hz, IR resolution of 80 × 60 pixels). Thermal images were captured after 20 s after the motor was started. Computer software (IR Thermal Imaging Application for FLIR E4) converted image 80 × 60 pixels to image 320 × 240 pixels. Operation of power tool with blocked air inlets yields increases in the temperature level (Figure 1).

Acquisition of thermal images is the first step of recognition of the state of the angle grinder. Measurement distance is equal to 0.4 m. The emissivity coefficient of the analyzed thermal image is equal to 0.6. The emissivity coefficient of steel elements should be 0.52–0.85. The emissivity coefficient of cold-plated steel (93 °C) is in the range of 0.75–0.85. The emissivity coefficient of steel surface hardened (200 °C) is equal to 0.52. The emissivity coefficient of stainless steel type 301 is in the range of 0.54–0.63.

**Figure 2:**
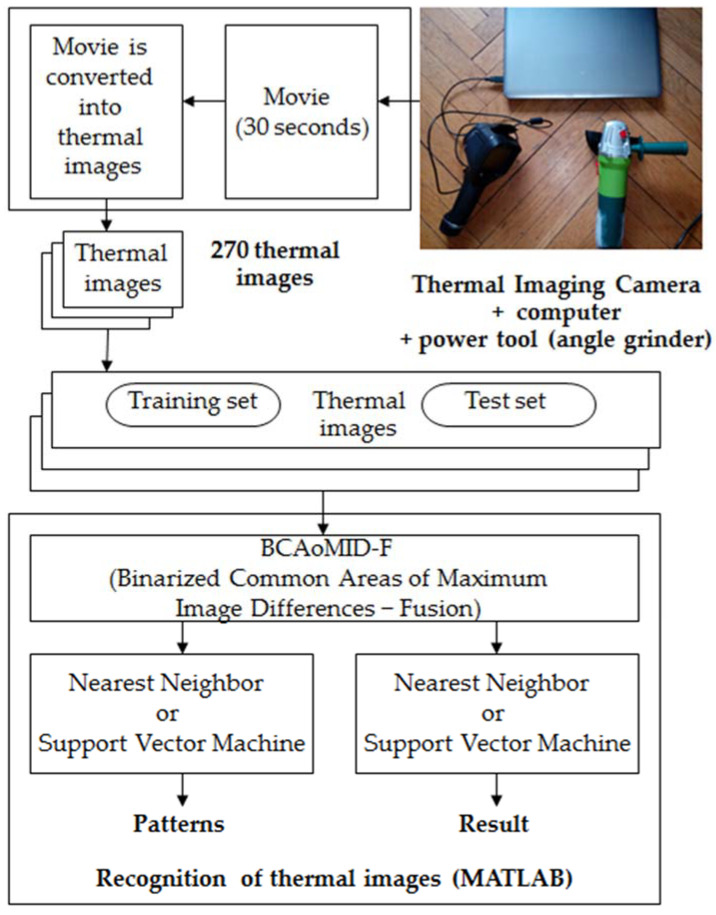
Processing and recognition of thermal images of the angle grinder.

Slight shifts of thermal imaging camera(+/−0.1 m) do not cause major changes for recognition results. Next, the captured movie is converted into thermal images. Thermal images are processed by the BCAoMID−F. Features of images are computed. Next, features are classified by the nearest neighbor classifier and Support Vector Machine. Processing and recognition of thermal images of the inspected angle grinder are shown in Figure 2. Matlab is used for the recognition and processing of thermal images.

## 4. Analyzed States of the Angle Grinder

**Figure 3:**
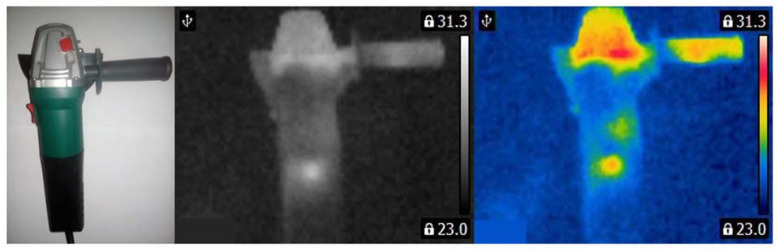
Healthy angle grinder.

**Figure 4:**
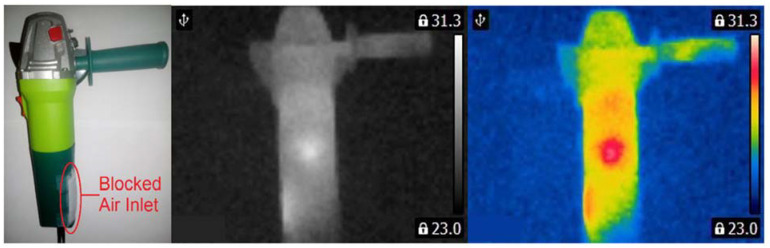
Angle grinder with 1 blocked air inlet.

**Figure 5:**
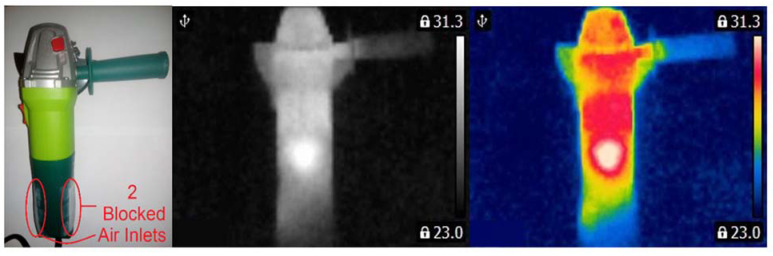
Angle grinder with 2 blocked air inlets.

Three angle grinders (Verto 51G053, 500 W) with different faults were used for measurements (Figure 3, Figure 4 and Figure 5). Measurements were carried out for the angle grinder. Thermal imaging camera captured different thermal images for each operating state of the angle grinder. The thermal images of the angle grinder were analyzed for the following cases: healthy angle grinder (Figure 3), angle grinder with 1 blocked air inlet (Figure 4), angle grinder with 2 blocked air inlets (Figure 5).

**Figure 6:**
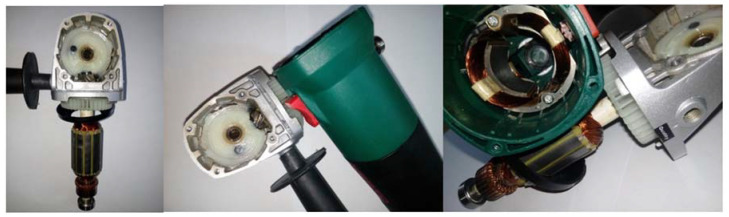
Parts of angle grinder (commutator motor, stator, fan, bearing).

We can notice that a healthy angle grinder has the lowest temperature. On the other hand, an angle grinder with 2 blocked air inlets has the highest temperature. Most heat is generated by the commutator motor (Figure 5). Parts of the angle grinder were shown in Figure 6.

### 4.1. BCAoMID−F (Binarized Common Areas of Maximum Image Differences−Fusion)

To extract features of thermal images, the author proposes the feature extraction method BCAoMID−F. The proposed method BCAoMID−F is based on differences between thermal images and comparison of obtained areas, histograms. This method is performed as follows:

1. Convert all thermal images into grayscale images (256 colors, value of the pixel is in the range of <0–1>).
2. Compute differences between training thermal images of the angle grinder: |**hag-ag1b|**, |**hag-ag2b|**, **|ag1b-ag2b|**, where **hag** − matrix (320 × 240) of the thermal image of the healthy angle grinder, **ag1b**−matrix (320 × 240) of the thermal image of the angle grinder with 1 blocked air inlet, **ag2b**−matrix (320 × 240) of the thermal image of the angle grinder with 2 blocked air inlets.
3. For computed differences |**hag-ag1b|**, |**hag-ag2b|**, **|ag1b-ag2b|**, set the proper value of threshold of binarization (t<|**hag-ag1b|**<u, for example: t = 0.1, u = 1).
4. Compute binary images (*binarization threshold* = 0.1).
5. Compute the sum of all binary images of the computed differences. It is denoted as matrix **S**.
6. Compute maximum value (*Max*) of the computed sum **S**.
7. Compute matrix **K** = **S**/*Max*.
8. Compute binary image−matrix **K**, where *z* < **K** < 1 (set 0 for values of matrix K less than *z*, set 1 for values of matrix K greater than *z*), *z* is set experimentally <0, 1>. For the analysis, the author set *z* = 0.5. Computed binary image **K** has values 0 and 1.
9. For each training and test thermal image denoted as matrix **B**, compute matrix **G** = **B** + **K**.
10. Compute matrix (**G** < 1.001) = 0 (set 0 for values of matrix **G** less than 1.001).
11. Compute matrix **V**, where **V** = **G** − 1
12. Compute three features: compute the sum of pixels of the matrix **V**, compute a histogram of the matrix **V**, compute PCA (Principal Component Analysis) of the matrix **V**.

**Figure 7:**
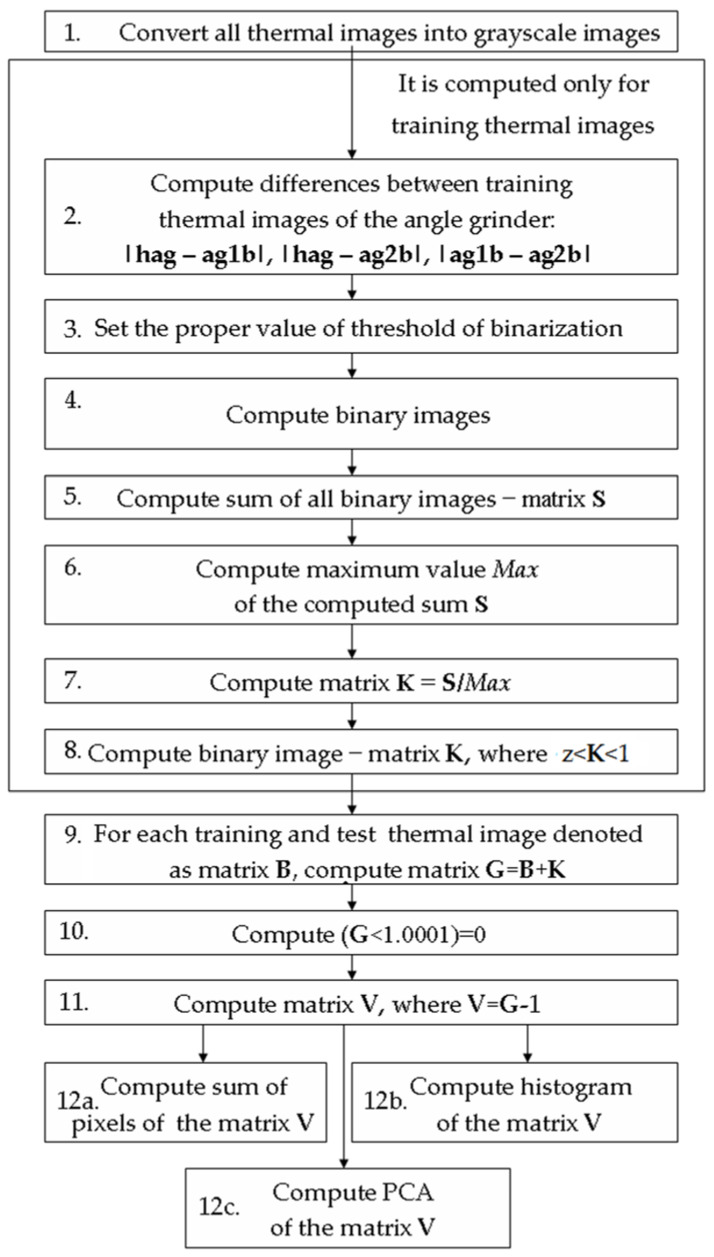
Flowchart of the feature extraction method BCAoMID−F.

**Figure 8:**
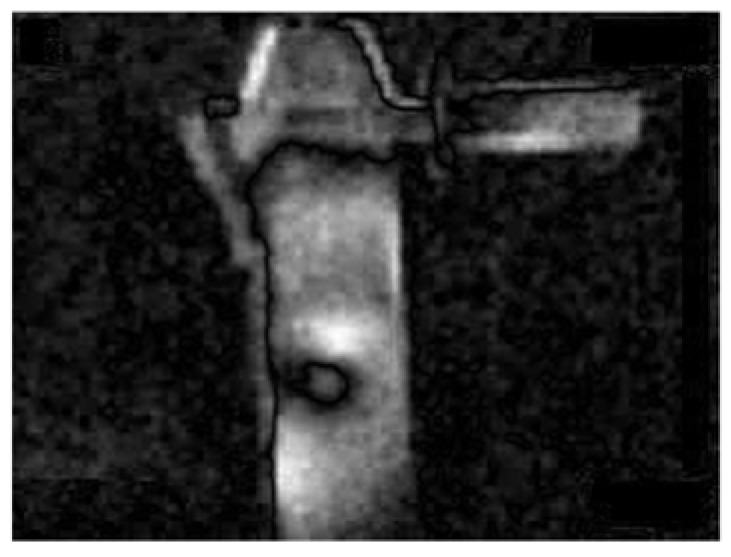
Difference of thermal images: |**hag-ag1b|**, where **hag**−matrix (320 × 240) of the thermal image of the healthy angle grinder, **ag1b**−matrix (320 × 240) of the thermal image of the angle grinder with 1 blocked air inlet.

**Figure 9:**
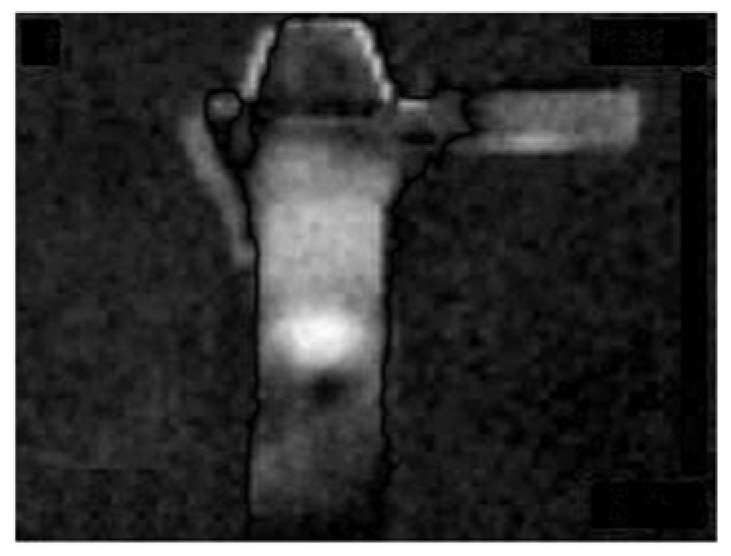
Difference of thermal images: |**hag-ag2b|**, where **hag**−matrix (320 × 240) of the thermal image of the healthy angle grinder, **ag2b**−matrix (320 × 240) of the thermal image of the angle grinder with 2 blocked air inlets.

**Figure 10:**
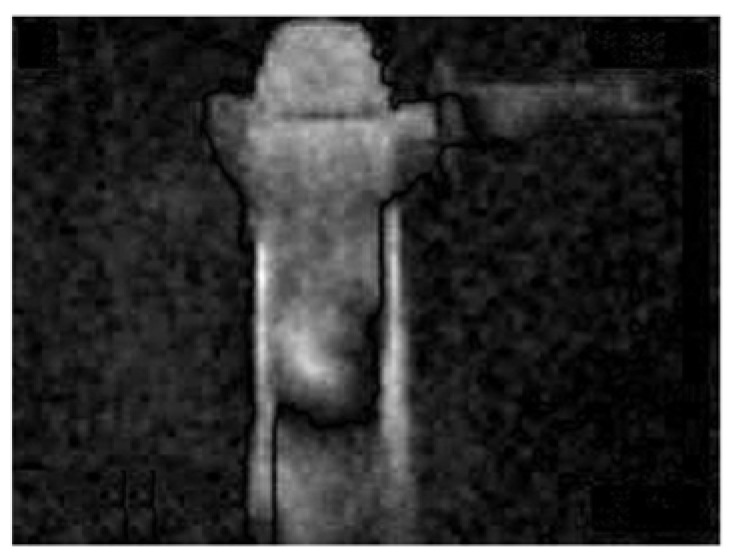
Difference of thermal images: |**ag1b-ag2b|**, where **ag1b**−matrix (320 × 240) of the thermal image of the angle grinder with 1 blocked air inlet, **ag2b**−matrix (320 × 240) of the thermal image of the angle grinder with 2 blocked air inlets.

A flowchart of the feature extraction method BCAoMID−F was presented in Figure 7. Computed differences between training thermal images of the angle grinder |**hag-ag1b|**, |**hag-ag2b|**, **|ag1b-ag2b|** are presented (Figure 8, Figure 9 and Figure 10).

**Figure 11:**
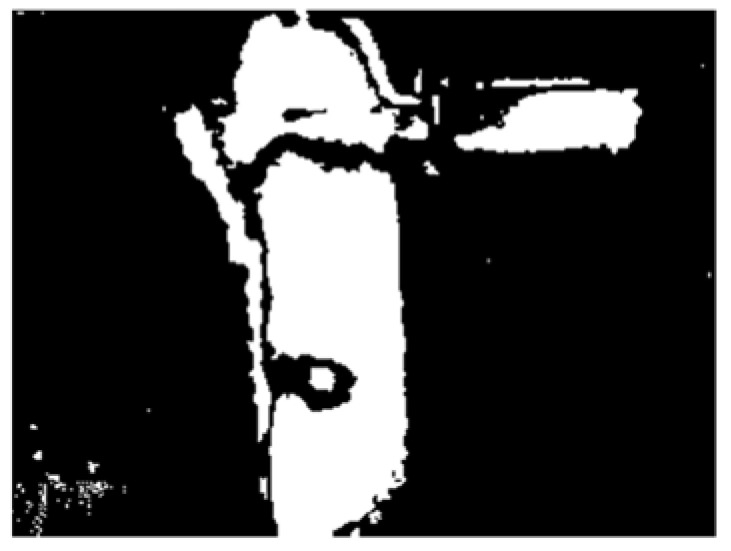
Binary image (0.1 < |**hag-ag1b|** < 1) of difference: |**hag-ag1b|**.

**Figure 12:**
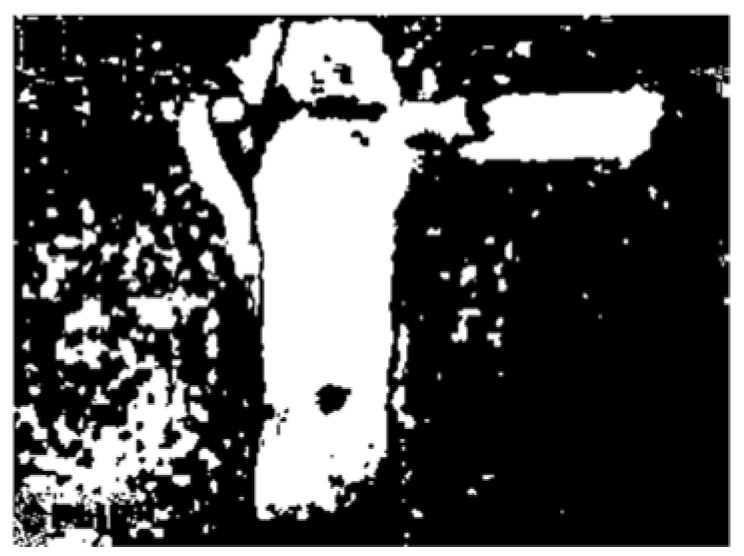
Binary image (0.1 < |**hag-ag2b|** < 1) of difference: |**hag-ag2b|**.

**Figure 13:**
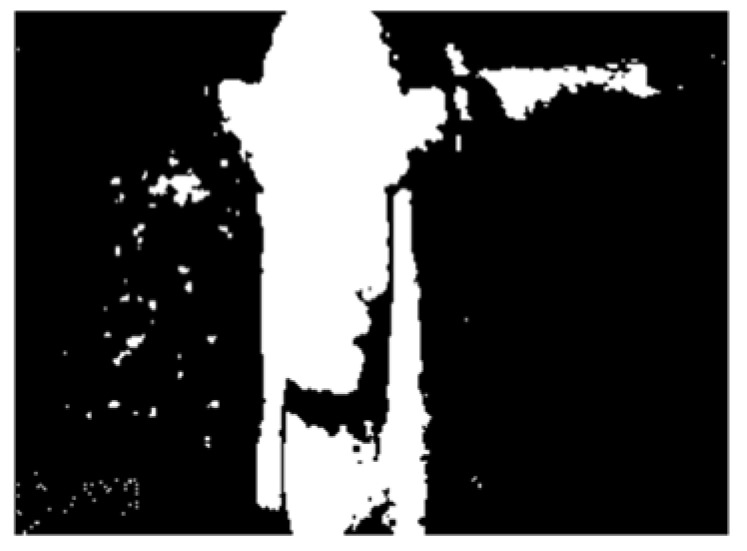
Binary image (0.1 < |**ag1b-ag2b|** < 1) of difference: |**ag1b-ag2b|**.

The next step was binarization of computed differences. Threshold was equal to 0.1 (0.1 < |difference| < 1). Figure 11, Figure 12 and Figure 13 show binary images for considered differences.

**Figure 14:**
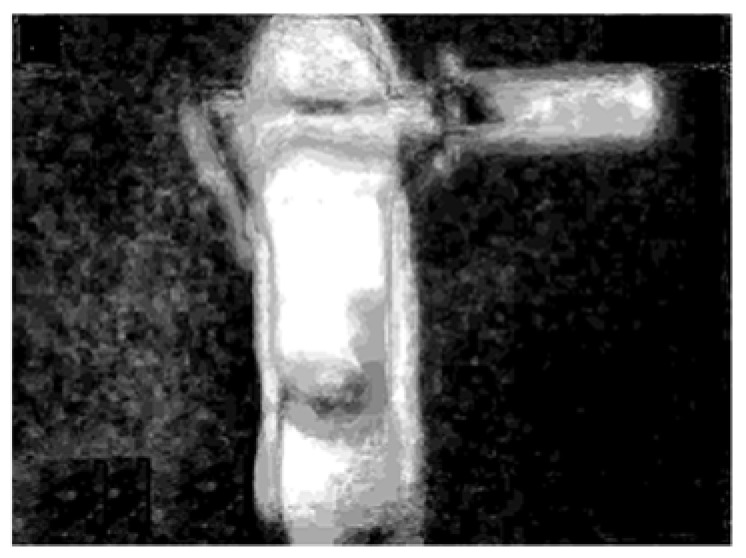
Computed matrix **K** for all training thermal images.

The computed matrix **K** for all training thermal images of the angle grinder is presented in Figure 14.

**Figure 15:**
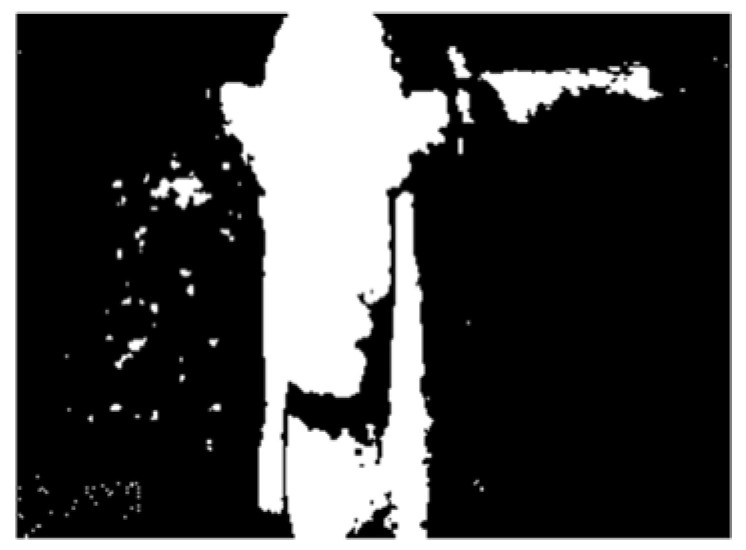
Binary image (0.5 < **K** < 1) of the matrix **K.**

The next, the step was binarization of matrix **K**. Binarization threshold was equal to 0.5 (0.5 < **K** < 1). It is shown in Figure 15.

**Figure 16:**
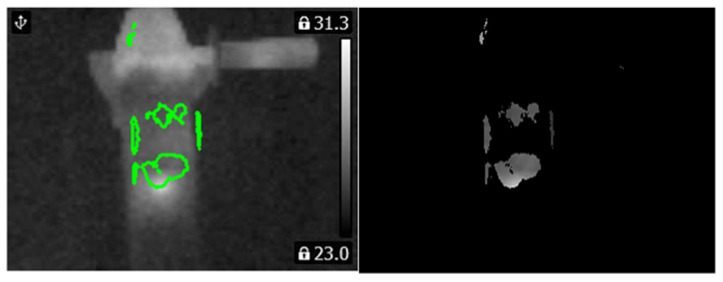
Values of the matrix **V** for the thermal image of the healthy angle grinder (indicated by the green line).

**Figure 17:**
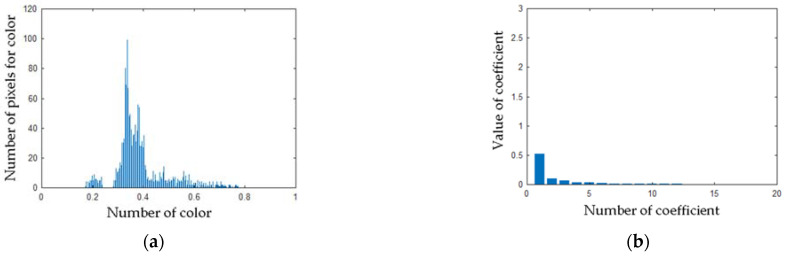
(**a**) Histogram of the matrix **V** for the thermal image of the healthy angle grinder (**b**) Values of PCA of the matrix **V** for the thermal image of the healthy angle grinder.

Computed matrix **V** for the healthy angle grinder was presented in Figure 16. Histograms and PCA values for the healthy angle grinder were presented in Figure 17.

**Figure 18:**
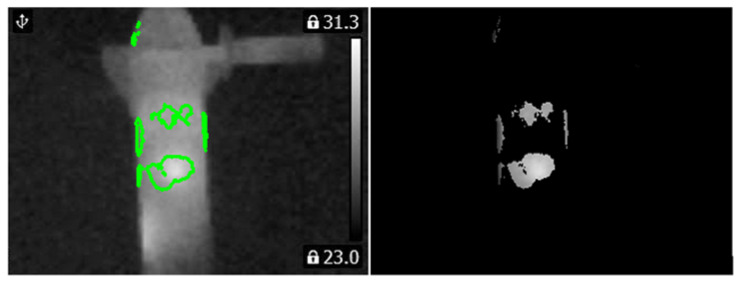
Values of the matrix **V** for the thermal image of the angle grinder with 1 blocked air inlet (indicated by the green line).

**Figure 19:**
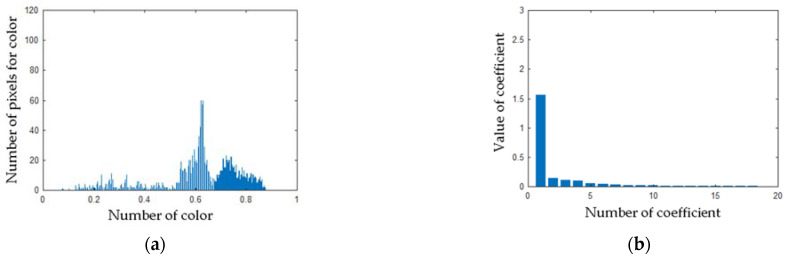
(**a**) Histogram of the matrix **V** for the thermal image of the angle grinder with 1 blocked air inlet (**b**) Values of PCA of the matrix **V** for the thermal image of the angle grinder with 1 blocked air inlet.

Computed matrix **V** for the angle grinder with 1 blocked air inlet was presented in Figure 18. Histograms and PCA values for the angle grinder with 1 blocked air inlet were presented in Figure 19.

**Figure 20:**
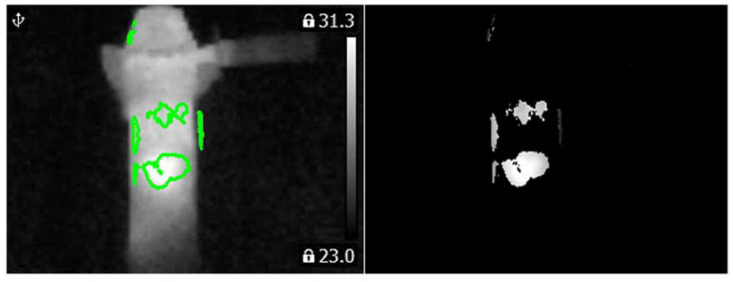
Values of the matrix **V** for the thermal image of the angle grinder with 2 blocked air inlets (indicated by the green line).

**Figure 21:**
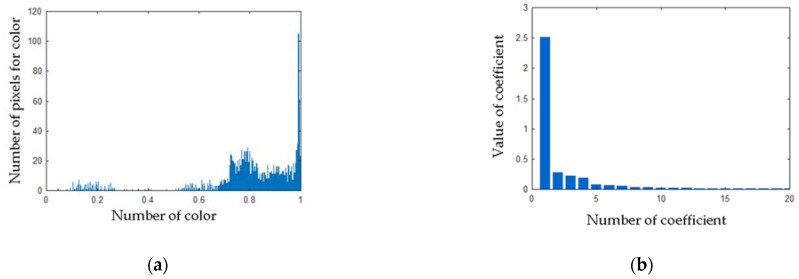
(**a**) Histogram of the matrix **V** for the thermal image of the angle grinder with 2 blocked air inlets (**b**) Values of PCA of the matrix **V** for the thermal image of the angle grinder with 2 blocked air inlets.

Computed matrix **V** for the angle grinder with 2 blocked air inlets was presented in Figure 20. Histograms and PCA values for the angle grinder with 2 blocked air inlets were presented in Figure 21.

The BCAoMID−F computed features of thermal images of the angle grinder. The author used the Nearest Neighbor classifier and Support Vector Machine for the classification of thermal images. We can notice that considered classes can be separated by a line/hyperplane. The classification methods such as the NN classifier and SVM classifier are proper for linearly separable features.

There are also other classification methods proper for linearly separable features. They are the following: neural networks [25,26,27], Naive Bayes [28], Linear Discriminant Analysis, fuzzy classifiers, adaptive neuro-fuzzy inference systems [29]. The author used a limited number of classification methods due to similar results.

### 4.2. Principal Component Analysis

The PCA (Principal component analysis) reduces the dimensionality of an original data set into a smaller output data set. Output data is uncorrelated. The PCA computes the principal components using a linear transformation. The principal components are eigenvectors of the covariance matrix. The matrix composed of eigenvectors can be converted into a new space to achieve dimensionality reduction of the original data set. More information about PCA is available in the literature [30,31,32].

### 4.3. Classification Using the Nearest Neighbor

One of the machine learning methods for classifying linearly separable data is the NN classifier. This method is simple and easy to implement. This classifier is widely used for economic forecasting, financial distress prediction models, data mining, pattern recognition, image recognition, signal recognition, text categorization, speaker recognition, climate forecasting, agriculture, financial modeling, analysis of glucose in the blood, genetic algorithms.

The steps of the NN classifier are following:

(1) load training and test feature vectors,
(2) set *k* = 1 (*k* − number of nearest neighbors),
(3) Compute the distance *d*, where d = Σ|**a**-**b**|, **a**—test feature vector, **b**—training feature vector,
(4) for all computed distances *d*, select the nearest distance,
(5) select the label of the predicted class.

The NN method is faster than other methods based on pattern recognition. The NN classifier has no training period. For a large number of training feature vectors, it does not work well. For the small number of training feature vectors, it is very fast and effective. Another advantage is that the new feature vector can be added to the training set easily. The advantage is that the NN classifier is not complex. It is also used for image classification successfully. There are also some disadvantages of the NN. For high-dimensional training feature vectors, it does not work well. The classifier is sensitive to noisy training feature vector. Sometimes outliers should be removed. The description of the NN classifier can be found in papers [33,34,35,36].

### 4.4. Support Vector Machine

The Support Vector Machine (SVM) is the method for data classification. This method of classification uses training and test feature vectors. It computed the margin of separation between classes and separating hyperplane. The SVM is commonly used for face recognition, text recognition, classification of images, fault diagnosis.

There are some advantages of the SVM. The SVM works very well if feature vectors are linearly separable. The classifier also works fast. It is a good classifier for low-dimensional feature vectors. For high-dimensional feature vectors, we need many feature vectors. The disadvantage of the classifier is that it does not work well for many overlapping classes. The SVM is also sensitive to noisy feature vectors. The description of the SVM classifier can be found in papers [37,38,39].

## 5. Results of the Analysis of Thermal Images

Recognition of thermal images was carried out for three states of the angle grinder: healthy angle grinder, angle grinder with 1 blocked air inlet, angle grinder with 2 blocked air inlets. Analyzed angle grinders had the following parameters: Rated power *RP* = 500 W, power supply frequency *f* = 50 Hz, power supply voltage *U* = 230 V, rated rotational speed *S* = 12,000 rpm, weight *W* = 1.64 kg. Measurements were carried out in a 3 m × 3 m room.

The author analyzed 60 training thermal images and 270 test thermal images. The analysis was based on the cross-validation method. The author used the following formula to compute recognition efficiency (1):

(1) $$R_{AG}=100\%*(RC_{AG})/(A_{AG})$$

where: *R_AG_*—recognition efficiency of a selected class of the angle grinder, *RC_AG_*—number of test thermal images recognized correctly, *A_AG_*—number of all test thermal images of a selected class of the angle grinder.

For considered classes of the angle grinder total recognition efficiency *TR_AG_* was defined as (2):

(2) $$TR_{AG}=(R_{AG1}+R_{AG2}+R_{AG3})/3$$

where *R_AG1_*−*R_AG_* for the healthy angle grinder, *R_AG2_*−*R_AG_* for the angle grinder with 1 blocked air inlet, *R_AG3_*−*R_AG_* for the angle grinder with 2 blocked air inlets.

Table 1, Table 2, Table 3, Table 4, Table 5 and Table 6 show recognition results for using the BCAoMID−F.

Values of *TR_AG_* were in the range of 98.5–100%. Values of *R_AG_* were in the range of 97.7–100%. The highest values of *TR_AG_* were computed for the Nearest Neighbor classifier (Table 1, Table 2 and Table 3). The lowest values of *TR_AG_* were computed for BCAoMID−F, PCA, and SVM (Table 6). We can notice, that features were extracted properly. NN and SVM also classified data successfully.

The proposed method BCAoMID−F can be used for other electrical devices for example gearboxes. Total recognition efficiency for analyzed states was similar to other methods using thermography [16,40,41]. The proposed method BCAoMID−F has some advantages. It is: fast, non-invasive, low-cost, slight shifts of thermal imaging camera(+/−0.1 m) do not cause major changes for recognition results. The limitation of the BCAoMID−F method is its application to selected devices generating high-temperature heat on surfaces for different faults.

## 6. Conclusions

This work described the thermal fault diagnosis method of the angle grinder. The proposed method used feature extraction method–BCAoMID−F, Nearest Neighbor classifier, and SVM. Three different corresponding cases of thermal images were considered: healthy angle grinder, angle grinder with 1 blocked air inlet, angle grinder with 2 blocked air inlets.

Total recognition efficiency for 3 classes (*TR_AG_*) was in the range of 98.5–100%. The presented approach was efficient for fault diagnosis of electrical devices such as power tools. The conclusions of the paper are following:

Analysis showed that the proposed method is useful for 3 different states of the angle grinder.

(1) Thermal images should be captured at the same distance (thermal imaging camera–analyzed motor).
(2) The proposed method is efficient for measurement distance equal to 0.4 m. Slight shifts of thermal imaging camera(+/−0.1 m) do not cause major changes for recognition results.
(3) Recognition rate for considered states was high (*TR_AG_* is in the range of 98.5–100%).
(4) The same conditions and equipment should be used for measurements. The temperature of air in a room should be similar for all training and test thermal images.
(5) Different types of motors can be diagnosed by the proposed method successfully.
(6) The proposed method is fast. It can be implemented as a condition online monitoring system.
(7) The method is non-invasive. It can be used for conditions of difficult access.
(8) The proposed method uses a computer and thermal imaging camera. The cost of the experimental setup is low (about 1400–1600$).

Future research will be focused on improvements of techniques of fault diagnosis based on signal analysis such as vibrations, acoustic signals, thermal signals. Various types of faults and machines will be analyzed. It will allow to development of improved methods of fault diagnosis of machinery.

## Figures and Tables

**Table 1:** Values of *TR_AG_* and *R_AG_* for using of BCAoMID−F, the sum of pixels, and NN classifier.

State of the Angle Grinder	*R_AG_* [%]
Healthy AG	100
AG with 1 blocked air inlet	100
AG with 2 blocked air inlets	100
	*TR_AG_* [%]
3 states of the AG	100

**Table 2:** Values of *TR_AG_* and *R_AG_* for using of BCAoMID−F, histogram, and NN classifier.

State of the Angle Grinder	*R_AG_* [%]
Healthy AG	100
AG with 1 blocked air inlet	100
AG with 2 blocked air inlets	100
	*TR_AG_* [%]
3 states of the AG	100

**Table 3:** Values of *TR_AG_* and *R_AG_* for using of BCAoMID−F, PCA, and NN classifier.

State of the Angle Grinder	*R_AG_* [%]
Healthy AG	100
AG with 1 blocked air inlet	100
AG with 2 blocked air inlets	100
	*TR_AG_* [%]
3 states of the AG	100

**Table 4:** Values of *TR_AG_* and *R_AG_* for using of BCAoMID−F, the sum of pixels, and SVM.

State of the Angle Grinder	*R_AG_* [%]
Healthy AG	100
AG with 1 blocked air inlet	100
AG with 2 blocked air inlets	100
	*TR_AG_* [%]
3 states of the AG	100

**Table 5:** Values of *TR_AG_* and *R_AG_* for using of BCAoMID−F, histogram, and SVM.

State of the Angle Grinder	*R_AG_* [%]
Healthy AG	100
AG with 1 blocked air inlet	100
AG with 2 blocked air inlets	100
	*TR_AG_* [%]
3 states of the AG	100

**Table 6:** Values of *TR_AG_* and *R_AG_* for using of BCAoMID−F, PCA, and SVM.

State of the Angle Grinder	*R_AG_* [%]
Healthy AG	100
AG with 1 blocked air inlet	97.7
AG with 2 blocked air inlets	97.7
	*TR_AG_* [%]
3 states of the AG	98.5

## Data Availability

Not applicable.
